# Draft Genome Sequence of *Ochroconis constricta* UM 578, Isolated from Human Skin Scraping

**DOI:** 10.1128/genomeA.00074-14

**Published:** 2014-04-17

**Authors:** Chai Ling Chan, Su Mei Yew, Shiang Ling Na, Yung-Chie Tan, Kok Wei Lee, Wai-Yan Yee, Yun Fong Ngeow, Kee Peng Ng

**Affiliations:** a Department of Medical Microbiology, Faculty of Medicine, University of Malaya, Kuala Lumpur, Malaysia; b Codon Genomics SB, Jalan Bandar Lapan Belas, Pusat Bandar Puchong, Selangor Darul Ehsan, Malaysia

## Abstract

*Ochroconis constricta* is a soilborne dematiaceous fungus that has never been reported to be associated with human infection. Here we report the first draft genome sequence of strain UM 578, isolated from human skin scraping. The genomic information revealed will contribute to a better understanding of this species.

## GENOME ANNOUNCEMENT

Members of the genus *Ochroconis* are dematiaceous fungi that have been reported to cause phaeohyphomycosis in immunocompromised and immunocompetent patients (1–4). However, the *Ochroconis constricta* species of *Ochroconis* has never been reported to have been isolated from clinical specimens or to cause any human infections to date. *O. constricta* is normally found in soil, considered to be a source of cutaneous fungal infections in humans and animals (5). We have isolated a dematiaceous fungus from human skin scraping, which was identified as *O. constricta* UM 578 by using both morphological and molecular approaches. Here, we report the first draft genome sequence of *O. constricta* UM 578.

The genome of *O. constricta* UM 578 was sequenced to 87-fold coverage using the Illumina HiSeq 2000 platform by combining both 500-bp (22,277,778 reads) and 5-kb (11,222,224 reads) DNA insert-size libraries. Velvet v1.2.08 (6), SSPACE 2.0 (7), and GapFiller v1.10 (8) were used for sequencing read assembly, scaffolding, and gap filling, respectively. The UM 578 genome is 34.6 Mb in size and arranged into 163 scaffolds (≥1,000 bp) with an *N*_50_ length of 1,172,353 bp and an overall G+C content of 52.1%. A total of 11,652 protein-coding genes were predicted in the UM 578 genome sequence by use of the combination of AUGUSTUS 2.5.5 (9), SNAP version 2006-07-28 (10), and Genemark-ES v2.3e (11) *ab initio* prediction software. Protein alignments were performed using exonerate v2.2.0 (12). All protein-coding genes were annotated against the NCBI nonredundant and Swiss-Prot databases. Gene ontology classification and KEGG metabolic pathway mapping were performed on all the predicted genes.

S12 (14 members), S10 (9 members), and A01A (8 members) were the three most abundant peptidase subfamilies present in the UM 578 genome sequence, according to a MEROPS database (13) search result. Both S12 and S10 are serine proteases. S12 family members are SE clan peptidases, which are specialized for involvement in bacterial cell wall metabolism, while S10 carboxypeptidases from the SC clan are synthesized as preproenzymes. The A01A subfamily members from the pepsin family are also preproenzymes, which carry out their activity in the lysosomal/endosomal system and have been suggested to function in the external digestion of food proteins by saprophytic organisms (13). To our knowledge, the draft genome sequence of *O. constricta* UM 578 is the first *Ochroconis* genome sequence deposited in the public database. The information generated from the *O. constricta* UM 578 genome will play an important role in further studies aimed at enhancing our understanding of this potentially opportunistic pathogen.

### Nucleotide sequence accession numbers.

The draft genome sequence of *O. constricta* UM 578 has been deposited in DDBJ/EMBL/GenBank under the accession no. AZYM00000000. The version described in this article is the first version, accession no. AZYM01000000.
